# Boosting textiles with plant extracts: an advanced sustainable antimicrobial innovation for direct skin applications

**DOI:** 10.1007/s13205-025-04475-3

**Published:** 2025-08-15

**Authors:** Cláudia S. Oliveira, Ashly Rocha, Jéssica Antunes, Augusta Silva, Carla Silva, Freni K. Tavaria

**Affiliations:** 1 Universidade Católica Portuguesa, CBQF - Centro de Biotecnologia e Química Fina – Laboratório Associado, Escola Superior de Biotecnologia, Rua Diogo Botelho 1327, 4169-005 Porto, Portugal; 2 Technological Centre of the Textile and Clothing Industries, CITEVE – Rua Fernando Mesquita 2785, 4760-034 Vila Nova de Famalicão, Portugal

**Keywords:** Plant extracts, Eucalyptus, Hop, Bioactive textiles, Antimicrobial textiles, Textile functionalization

## Abstract

This study investigates the incorporation of natural plant extracts, particularly eucalyptus and hop, into knitted fabrics to develop antimicrobial textiles. The antibacterial activity of the functionalized fabrics was evaluated against two pathogenic bacteria (*Staphylococcus aureus* and *Escherichia coli*) and one common skin commensal (*Staphylococcus epidermidis*). Biocompatibility with human skin cells was assessed through MTT assays, TO/PI, and Calcein/PI staining. Results demonstrated strong antimicrobial activity of the functionalized textiles against *S. aureus* and *S. epidermidis*, with less evident effects on *E. coli*. Fabrics, functionalized with hop extracts, demonstrated biocompatibility in the applied two-dimensional models; however, confirming their safety for human use requires further evaluation using advanced models and additional endpoints, such as skin sensitization and genotoxicity. Overall, the study highlights the potential of incorporating natural plant extracts in textile functionalization to create eco-friendly and antimicrobial materials that meet growing market demands for safety and sustainability. Future research is warranted to optimize performance and applications.

## Introduction

The market for antimicrobial textiles is expanding and requires innovative products to support the technological advancement of a modern society. This growing demand spans a wide range of industries, including: (i) *food industry,* where such products are essential for uniforms, gloves, and surface coverings to prevent food contamination; (ii) *healthcare institutions* where similar products are used to reduce the spread of infections; (iii) *consumer products* (everyday items such as clothing, sportswear, military uniforms, and home textiles) that benefit from improved hygiene, durability, and odor control, and (iv) *dermato-therapeutic strategies,* where antimicrobial textiles can aid in the treatment of skin-related infections. It is important to emphasize that most of these products are in direct contact with the human skin.

To meet the growing demand for antimicrobial textiles, various products have been successfully developed (Vakayil et al. 2021; Borkow et al. 2010; Wang et al. 2020; Roman et al. 2020; Lopes et al. 2015; Raeisi et al. 2021; Gauger et al. 2003; Wiegand et al. 2013). However, many of these rely on synthetic components such as metals (silver, copper, zinc, cobalt, and titanium, and chemical agents like quaternary ammonium compounds and triclosan (Vakayil et al. 2021; Barani 2014; Ravindra et al. 2010; Roman et al. 2020; Gauger et al. 2003). Given this reliance on synthetic materials, there is an urgent need to prioritize the incorporation of natural compounds as antimicrobial agents. Although natural compounds are often considered safer than the synthetic components, which may have undesirable side effects despite their beneficial properties, many natural agents can also pose health and environmental risks.

Plant extracts are recognized as rich sources of several bioactive compounds with antimicrobial properties, making them an important alternative for the textile industry (Kramar et al. 2021). For instance, extracts from eucalyptus and hop have demonstrated strong antimicrobial activity against both Gram-positive and Gram-negative bacteria in several in vitro studies (Elansary et al. 2017; Rasool et al. 2018; Kramer et al. 2015; Kolenc et al. 2022). The antimicrobial activity of *Eucalyptus spp*. has numerous medicinal applications, primarily due to the presence of bioactive compounds such as saponins, tannins, steroids, flavonoids, phenolic acids, and cardiac glycosides, which are typically found in extracts from the bark and leaves (Aleksic Sabo and Knezevic 2019; Elansary et al. 2017).

Hop (*Humulus lupulus*) is widely used in the brewing industry due to its bitterness, flavor aroma, stability, and antimicrobial protection of beer (Kolenc et al. 2022). The phytochemical screening has identified alpha acids (humulones), beta acids (lupulones), and flavonoids (i.e., xanthohumol) as the main compounds responsible for exerting the antimicrobial activities (Karabín et al. 2016; Knez Hrncic et al. 2019).

In this regard, we aimed to evaluate the potential use of plant extracts with strong in vitro antimicrobial activity as natural antibacterial agents for the development of antimicrobial textile materials. To achieve this, we functionalized a knitted fabric with extracts from eucalyptus and hop and explored the antimicrobial activity of the functionalized textiles against two pathogenic bacteria (*Staphylococcus aureus* and *Escherichia coli*) as well as a common skin commensal (*Staphylococcus epidermidis*). In addition, we assessed the biocompatibility of the functionalized knitted fabrics in human skin cells to ensure their safety for direct skin applications.

## Materials and methods

### Plant extraction

#### Eucalyptus

The mechanical processing of eucalyptus branches and leaves was carried out using a blade mill (SM 300 Retsch), which reduced the material size to 4 mm. Subsequently, 20% (m/v) *(*where m/v denotes mass/volume, corresponding to grams of powder per 100 mL of solution) of the eucalyptus branches were immersed in an aqueous solution of 0.1 M NaOH with 20% ethanol. The extraction process was performed using the Labomat BFA Mathis at 50 °C, with the temperature raised to 50 °C from room temperature (RT) at a gradient of 3 °C/min. After reaching 50 °C, the samples were extracted at this temperature for 60 min. After this time, the extracts were removed from the Mathis vessels, left to cool down to RT, and filtered under vacuum pressure, yielding a brown filtrate. This filtrate was collected in Schott flasks and stored at 4 °C until further use.

#### Hop

The mechanical processing of hop was carried out using a ball mill, which reduced the residue to powder. Subsequently, 20% (m/v) of hop powder was immersed in an aqueous solution of 0.3 M NaOH with 20% ethanol. The extraction process was performed using the Labomat BFA Mathis at 50 °C, with the temperature raised to 50 °C at heating gradient of 3 °C/min for 60 min at 30 rpm. After extraction, the mixture was cooled to RT and filtered under vacuum pressure, yielding a brown filtrate. This filtrate was collected in Schott flasks and stored at 4 °C until further use.

### Functionalization of textiles with extracts of hop and eucalypt

A navy-blue knitted fabric composed of 95% lyocell ad 5% elastane (170 g/m^2^) was supplied by Impetus, Portugal. The entire process of functionalizing the textile materials with the plant extracts under study was carried out by Technological Centre of the Textile and Clothing Industries (CITEVE, Portugal). To eliminate any contaminants, the fabric was washed at 60 °C for 60 min in a domestic laundry machine, using a non-ionic detergent at a concentration of 1 g/kg of textile. Before functionalization, the fabric was pre-treated by cationization to modify the surface electric charge of the textile fibers, introducing positively charged sites (cationic groups). This step is crucial to enable the subsequent adherence of the extract to the knitted fabric.

**Scheme 1:**
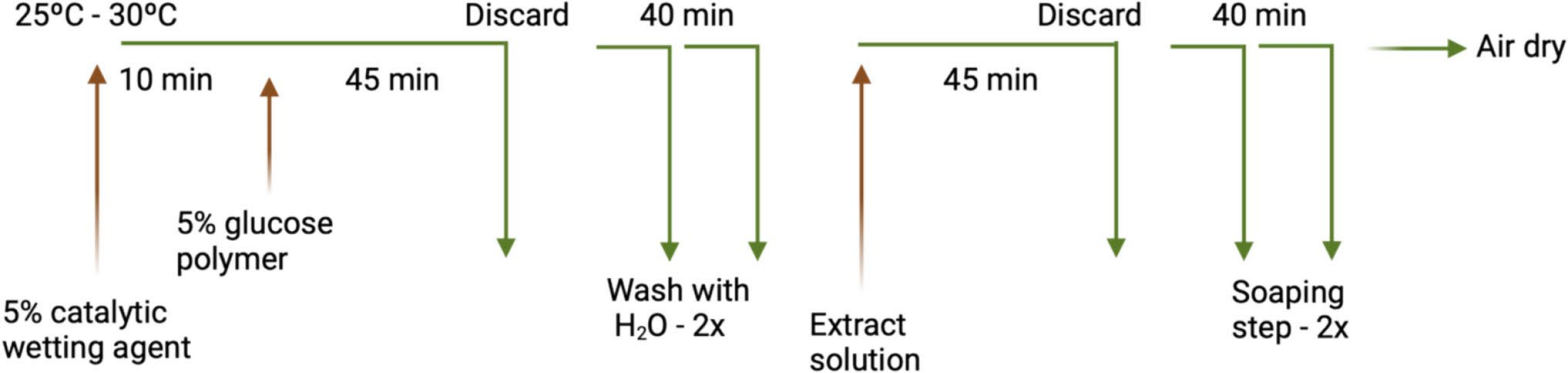
Exhaust procedure used for the cationization and functionalization of the knitted fabrics

For this, the cationization of the knitted fabrics was performed using the exhaust method with a Labomat BFA Mathis (procedure depicted in Scheme 1). Initially, under the conditions specified by the supplier, an aqueous solution containing 5% AquiNat Set (provided by Aquitex) based on the fabric weight (ofw) was applied to the fabric in stainless steel Labomat cups at 30 °C for 10 min at 30 rpm, with a heating gradient of 3 °C/min. Subsequently, 5% AquiNat Plus based on the fabric weight (ofw), as per the supplier’s instructions, was added to the solution, and the treatment was continued at 30 °C for 45 min at 30 rpm, maintaining the same heating gradient. Then the solution was discarded, and the fabric was washed with water at 30 °C for 20 min at 30 rpm, repeated twice, each with a heating gradient of 3 °C min^−1^.

The functionalization treatment of the knitted fabric involved using a liquor ratio 1:10 of the extract solution (Eucalyptus or Hop) at 30 °C for 45 min at 30 rpm, with a heating gradient of 3 °C min^−1^, followed by discarding the extract solution (Scheme 1). Subsequently, a soaping step was performed to remove any unfixed solution or active principles using Tanapal Pura (an anionic soaping agent—0.5 g/kg of textile, provided by Grupo ADI) at a liquor ratio 1:10 at 30 °C for 20 min at 30 rpm, repeated twice, each with a heating gradient of 3 °C min^−1^. Finally, the samples were rinsed with water, squeezed, and air dried.

The different conditions were identified as follows: (1) cationized knitted fabrics without functionalization with any plan extract (Textile control); (2) cationized and functionalized knitted fabric with eucalyptus extract; and (3) cationized and functionalized knitted fabric with hop extract. Afterward, to conduct all the assays and prevent contamination, the knitted fabrics were cut into round-shaped discs (± 1.5 cm in diameter) and sterilized using UV light for 20 min on each side.

### Antimicrobial assays

#### Bacterial strains

*Staphylococcus aureus* (ATCC 6538), *Staphylococcus epidermidis* (DSH 20044), and *Escherichia coli* (ATCC 8739) suspensions were prepared in Tryptic Soy Broth (TSB, Biokar Diagnostics). They were then allowed to incubate at 37 °C for 24 h. A small sample was extracted from the resulting bacterial suspension using a sterile loop and streaked onto Tryptic Soy Agar (TSA, Biokar Diagnostics). The bacteria were then incubated under optimal conditions until visible colonies formed. Subsequently, a single isolated colony was selected, inoculated in TSB and incubated at 37 °C for approximately 24 h. Fresh inocula for each bacterial strain were prepared from the previous inoculum for all assays. The bacterial concentration (CFU/mL) of each inoculum was determined during the mid-log growth phase using densitometry and CFU counting for the antimicrobial assays.

#### Antimicrobial qualitative assay

The antibacterial testing procedure followed ISO 20645:2004 guidelines with slight modifications. Briefly, 1 mL of each bacterial inoculum (concentration ~ 8 log CFU/mL) was added to a flask containing 150 mL of TSA heated to 45 °C. To cultivate a bacterial lawn, 5 mL of the bacterial suspension was evenly spread over 10 mL of pre-solidified TSA. After 10–15 min, three textile pieces/per condition were aseptically placed on the agar surface using sterile tweezers. The agar plates were then incubated at 37 °C for 2 and 24 h. After 24 h, bacterial growth around and beneath the textile samples was assessed according to International Organization for Standardization (ISO) 20645:2004 standards. Each condition was tested in triplicate.

#### Evaluation of the number of viable bacteria attached to the textile materials

**Fig. 1:**
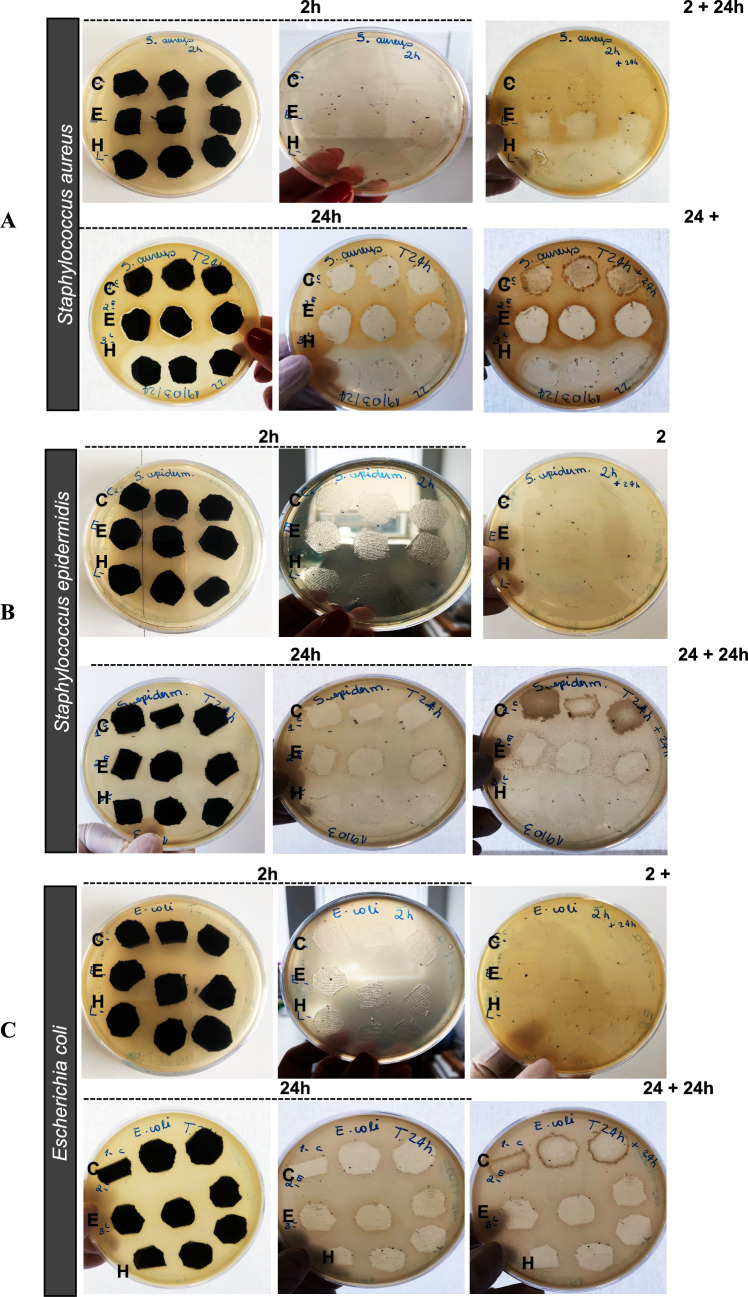
Results of the qualitative antimicrobial assay (agar diffusion test) performed in **A** *S. aureus*, **B** *S. epidermidis*, and **C** *E. coli*. TSA plates containing bacteria inoculum and functionalized knitted fabrics after 2 and 24 h of contact are shown. Plates on the left (before the removal of the textile samples) show the inhibition zone around the textile specimens after 2 and 24 h of direct contact with the different textile samples. Plates at the center show bacterial growth under the samples. Plates on the right are incubated for more than 24 h in the absence of textile specimen. The knitted fabrics are classified as **D** textile control; **E** knitted fabric functionalized with eucalyptus extract; and **H** knitted fabric functionalized with hop extract

To evaluate the bacteria adhesion onto the textile materials, we used the methodology of Ivankovic et al. (2022) with some modifications (Fig. 1a). Briefly, the same methodology was performed for the antimicrobial qualitative assays (described above was used). Following 2 and 24 h of incubation, each textile sample was retrieved from the agar plate and immersed in 2 mL of sterile Ringer solution (Biokar Diagnostics). The samples were then vortexed five times for ± 5 s to detach bacterial cells from the textiles. Subsequently, the supernatant was serially diluted up to 10 ^−5^ and plated onto TSA. Following another 24 h of incubation at 37 °C, the colonies were counted, and the bacterial count was expressed as log CFU/mL.

### Skin cell line and cytotoxicity assays

The immortalized human keratinocyte line (HaCaT) sourced from Cell Lines Service (Oppenheim, Denmark) was thawed and cultured in Dulbecco’s modified Eagle medium (DMEM, high glucose) supplemented with 10% fetal bovine serum (FBS, BioWest) and 1% penicillin–streptomycin–Fungizone solution (Pen-strep, Lonza). The keratinocyte cell line was cultured in T75 flasks at 37 °C in a 5% CO_2_ humidified atmosphere throughout the entire experimental duration.

#### Cytotoxicity assays

To conduct the metabolic activity and cell viability assays, keratinocytes were seeded onto a 24-well plate at a density of 1 × 10^5^ cells/mL and allowed to adhere overnight. Subsequently, the culture media was replaced, and a textile sample (± 1.5 cm in diameter) was placed onto the cell monolayer in each well to ensure direct contact exposure (Fig. 3a). In addition, cells cultured without fabric served as the negative control and cells treated with 10% of dimethyl sulfoxide (DMSO) were used as the positive control Following a 24-h incubation period, the following different assays were performed to evaluate the cell viability of keratinocytes after a direct contact with the different textiles.

##### Metabolic activity of keratinocytes

The metabolic activity of keratinocytes was assessed using the 3-(4,5-dimethylthiazol-2-yl)-2,5-diphenyltetrazolium bromide (MTT) assay. The culture media was replaced with 450 µL of DMEM and 50 µL of MTT solution (5 mg/mL) and incubated for 3–4 h at 37 °C in a CO_2_ incubator. After the incubation, the media was aspirated, and 500 µL of DMSO per well was added to dissolve the formazan crystals. The plates were then agitated for 10 min at room temperature. Absorbance (Abs) was measured at 570 nm using a microplate reader (Synergy 4, Biotek). Cell viability percentage was calculated as follows:

$${\text{Cell viability percentage }} = \frac{{{\text{Abs of treated cells}} \times 100}}{{{\text{Abs of control cells}}\,\left( {\text{cells cultured without fabric}} \right)}}$$

Each sample was tested in triplicate in a single experiment conducted in three independent assays.

##### Quantification of viable, damaged, and non-viable keratinocytes

To understand the results of MTT assay, the BD™ Cell Viability Kit (BD Biosciences, USA) was used to quantify viable, damaged and non-viable keratinocytes following direct exposure to the different textiles under investigation. After 24 h of contact, cell culture supernatants were collected, and the remaining adherent cells were washed with PBS, trypsinized, and centrifuged. The resulting cell pellets were resuspended in 500 μL of PBS, stained with 4 μL of thiazole orange (TO) and 2 μL of propidium iodide (PI), and incubated at room temperature (RT) for 5 min according to the manufacturer’s instructions.

The samples were analyzed immediately using a BD Accuri™ C6 flow cytometer (BD Biosciences, Franklin Lakes, NJ, USA). A dot plot with FL1 (TO detection) and FL3 (PI detection) channels enabled discrimination between live cells (TO^+^/PI^−^), damaged cells (TO^+^/PI^+^), and non-viable cells (TO^−^/PI^+^), following the BD™ Cell Viability Kit protocol. Each condition was assessed in triplicate across three independent experiments.

##### Live and dead assay

To further complement these findings, we conducted a live/dead assay to evaluate the viability of keratinocytes following direct exposure to the textile samples. Cells were plated and treated similarly to the previous cytotoxicity assays. However, after 24 h of contact with the textile samples, the keratinocytes monolayers were washed with PBS washing and incubated with 5 μL of Calcein-AM (1 mg/mL Solution) and 2 μL of propidium iodide (PI, 50 μg/mL) in 1000 μL of PBS at 37 °C in a 5% CO2 humidified atmosphere for 20 min. Subsequently, the cells were washed with PBS, kept in PBS, and promptly observed under fluorescence microscopy (Axio Imager 2, Zeiss).

### Statistical analysis

Data are presented as means ± standard deviation of the mean and were analyzed using the GraphPad Prism 10 software. When appropriate, multiple comparisons were performed using the one-way ANOVA or two-way ANOVA. Statistical significant differences were considered as follows: *p < 0.05, **p < 0.01, ***p < 0.001, ****^,++++^*p* < 0.0001.

## Results and discussion

### Evaluation of the antibacterial potential of the knitted fabrics functionalized with eucalyptus and hop extracts

First, we assessed the antibacterial effect of the three different knitted fabrics against *S. aureus*, *S. epidermidis*, and *E. coli* according to the ISO ISO:20645:2004 standard (Table 1). These bacterial species were chosen to evaluate the effect of functionalized textiles with plant extracts on both pathogenic and non-pathogenic bacteria commonly found on human skin. *S. aureus* and *E. coli* are Gram-positive and Gram-negative bacteria, respectively, recommended as test microorganisms in the ISO: 20645:2004. However, we also included another Gram-positive bacterium, *S. epidermidis*, a key resident of the human skin microbiota.

**Table 1:** Assessment of antibacterial efficacy of textile materials in study according to the ISO 20645:2004 “Agar diffusion plate test” after 24 h of incubation

Bacteria	Sample	Growth under the specimen	Assessment of antibacterial efficacy according to ISO 20645:2004
	Textile control	Moderate	Insufficient effect
*S. aureus*	Textile functionalized with eucalyptus extract	None	Good effect
ATCC 6538			
	Textile functionalized with hop extract	None	Good effect
	Textile control	Moderate	Insufficient effect
*S. epidermidis*	Textile functionalized with eucalyptus extract	None/slight	Good effect
DSH 20044			
	Textile functionalized with hop extract	None	Good effect
	Textile control	Slight/moderate	Insufficient effect
*E. coli*	Textile functionalized with eucalyptus extract	Slight/moderate	Insufficient effect
ATCC 8739			
	Textile functionalized with hop extract	Slight/moderate	Insufficient effect

The results were interpreted using the ISO 20645 assessment after 24 h by observing the presence or absence of an inhibition zone around the textile sample and the bacterial growth under the textile (Table 1). In addition, we analyzed bacterial growth after 2 h of contact with the different textile samples. No visible growth was observed for any of the tested bacteria after 2 h of incubation (Fig. 1a). Interestingly, after incubating these plates for 24 h in the absence of textile samples, growth of *S. aureus* was observed at the location of the textile control, whereas complete growth inhibition was noted at the location of textiles functionalized with eucalyptus extract. Furthermore, textiles functionalized with hop extract showed a clear inhibition zone indicating an absence of bacterial growth. However, this behavior was not observed for *S. epidermidis* and *E. coli* after 2 h of contact with the textile samples and 24 h of incubation in the absence of textile specimens (Fig. 1b, c).

The evaluation of the plates after 24 h in the presence of textile specimens revealed notable results regarding the antibacterial effects of these materials. For *S. aureus*, no inhibition zone was observed around the textile control (Fig. 1a). However, a slight inhibition zone was noted around textiles functionalized with eucalyptus extract, and a substantial inhibition zone was observed around those functionalized with hop extract. Furthermore, while moderate growth was found under the textile control specimens, no growth was observed under either of the textiles functionalized with plant extracts. Moreover, after 24 h of incubation in the absence of textiles, evident growth was observed at the locations of the textile controls, whereas no growth was noted at the locations of textiles functionalized with eucalyptus and hop extracts. These results demonstrate the great antibacterial effects of these textiles against *S. aureus*.

Similarly, *S. epidermidis* showed comparable effects, though the inhibition zones for textiles treated with eucalyptus and hop were less pronounced due to the bacteria’s white coloration on TSA after 24 h of contact with the textile samples (Fig. 1b). These findings highlight the effective antibacterial properties of textiles functionalized with eucalyptus and hop against both Gram-positive bacteria.

In contrast, for *E. coli*, there was no significant visual difference in bacterial growth between the control textiles and those treated with eucalyptus and hop after both 2 and 24 h of incubation (Fig. 1c). In this case, and according to ISO 20645:2004 recommendations for interpretation, the functionalized knitted fabrics under study exhibit an insufficient effect against *E. coli* (Table 1).

To complement these results, we aimed to evaluate the amount of attached and viable bacteria on the knitted fabrics under study. This was necessary because the interpretation of the qualitative antimicrobial test according to ISO 20645:2004 can be somewhat subjective. In addition, some studies have reported that even control textiles without any antimicrobial agents may show “no growth” under the specimen when tested by the agar diffusion test after 24 h of incubation (Ivankovic et al. 2022). This usually happens because bacterial cells can firmly attach to textile materials, including wool and various cotton samples, forming a biofilm during multiplication (Ivankovic et al. 2022). Consequently, our research questions were: can knitted fabrics functionalized with plant extracts effectively inhibit bacterial growth over time? In addition, what differences exist between control textiles and those functionalized with plant extracts?

**Fig. 2:**
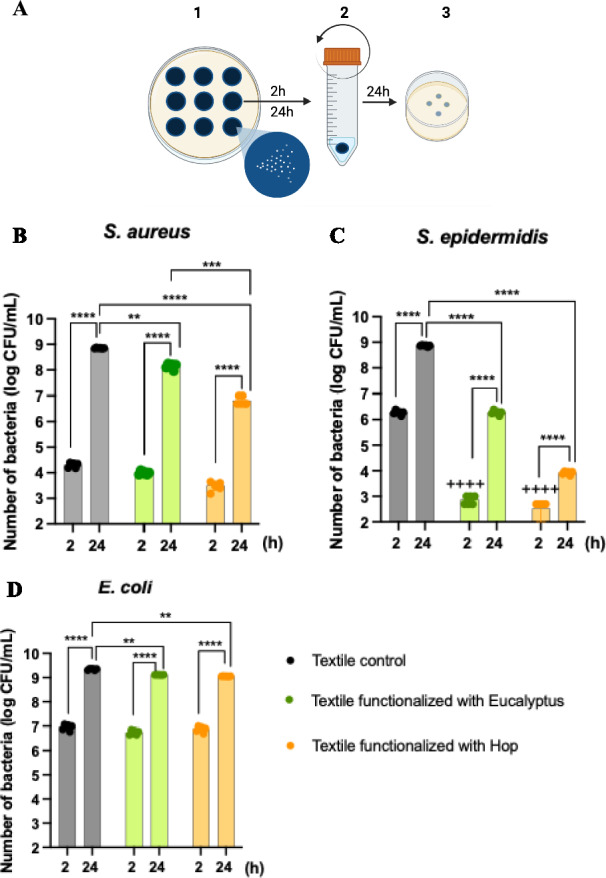
**A** Experimental design for the determination of the number of viable and attached bacteria on textile specimens after 2 and 24 h of contact with inoculated agar. **1** After incubation at 37 °C, bacterial growth around and under the different textile samples was assessed. **2** Each textile sample was then removed, transferred to 2 mL of sterile saline solution, and vortexed to detach the bacteria from the textile samples. **3** The supernatant was serially diluted and plated. The number of bacterial colonies was counted after incubation (37 ºC for 2 and 24 h). The number of viable **B** *S. aureus*, **C** *S. epidermidis*, and **D** *E. coli* adsorbed onto the textile specimens after 2 and 24 h of contact in the agar diffusion test are represented. Data are reported as the mean of three textile sample/condition. Statistically significant differences: **p < 0.01, ***p < 0 .001, and ****^,++++^*p* < 0.0001, where + indicate statistical significance with respect to the control (2 h)

To address these questions, we further investigated the agar diffusion results to understand the interaction between bacteria and different knitted fabrics, without and with plant extracts functionalization. This involved counting the viable cells attached to the textiles after 2 and 24 h of contact with the textile specimens in TSA inoculated with bacteria (Fig. 2a).

According to ISO 20645:2004, we observed no visible bacterial growth on any of the knitted fabrics after 2 h of contact. However, over time, *S. aureus*, *S. epidermidis*, and *E. coli* attached to the textiles during the initial 2 h and continued to multiply until 24 h, as indicated by the significant increase in bacterial numbers (*p* < 0.0001) (Fig. 2b–d). Notably, at both time points, Gram-positive bacteria attached significantly less to textiles functionalized with Eucalyptus and hop extracts compared to the control. It is important to note that, although both Gram-positive bacteria showed an increased number of viable cells from 2 to 24 h, their counts were significantly reduced compared to the textile controls at both time points (Fig. 2b, c). Furthermore, *S. epidermidis* exhibited greater sensitivity to the knitted fabrics functionalized with eucalyptus and hop extracts compared to *S. aureus*.

Regarding *E. coli*, the results were similar to those obtained with ISO 20645:2004, where we found no visual difference between the effects of the textile controls and the functionalized knitted fabrics with plant extracts. However, compared to the controls at 2 and 24 h of contact, there was a slight decrease in the number of attached/viable *E. coli* (Fig. 2d). These results suggest that knitted fabrics functionalized with eucalyptus and hop extracts have some antibacterial effect on Gram-negative bacteria, though this effect was not visually detectable and did not meet with the detection standards of ISO 20645:2004.

The comparison of our results with the existent literature was challenging for several reasons: (i) studies on antimicrobial textiles functionalized with natural components, specifically plant extracts, remain scarce, as most research prioritizes the incorporation of chitosan or essential oils (Szadkowski et al. 2024; Kramar et al. 2021; Gomes et al. 2013; Lopes et al. 2015; Stan et al. 2019; Costa et al. 2022); (ii) most research in this field employs qualitative assays to evaluate the antimicrobial activity, typically using agar diffusion tests to measure bacterial growth inhibition around and under the textile sample (Szadkowski et al. 2024; Zaharia et al. 2020); (iii) few studies investigate the ability of bacteria to adhere and grow on the textile specimen itself, rather than solely examining bacterial growth on the agar plate (Ivankovic et al. 2022); and (iv) studies have primarily focused on the antimicrobial activity against common skin pathogenic bacteria, often neglecting to evaluate the effect of antimicrobial textile materials on key resident members of the human skin microbiota, such as *S. epidermidis* (Kramar et al. 2021).

In addition, several approaches have been proposed to enhance the antibacterial properties of fabrics through various pre-treatments for textile surface modification. These approaches include chemical and natural polymer coatings, stabilization of metal nanoparticles, and surface plasma treatment (Szadkowski et al. 2024; Barani 2014; Naebe et al. 2022; Ravindra et al. 2010; Shahidi et al. 2007; Stan et al. 2019). In fact, pre-treating fabrics with chemical or biopolymer coatings have emerged as a promising and straightforward strategy for developing textiles with high antimicrobial activity. Similarly to our cationized knitted fabrics functionalized with eucalyptus and hop, cotton fabrics coated with chitosan and plant extracts, namely aloe vera, and essential oils, i.e., cinnamon, have been shown to effectively inhibit the growth of both Gram-positive and Gram-negative bacteria (specifically, *S. aureus* and *E. coli*) as evaluated by the agar diffusion test (Szadkowski et al. 2024). This work demonstrated a synergistic effect of plant extracts and essential oils in chitosan-coated cotton, enhancing the antibacterial properties of the chitosan coating formulation. Likewise, Stan et al. (2019) highlighted the antibacterial activity of 100% cotton and 50% cotton/50% polyester fabrics that have been cationized and functionalized with microcapsules containing sage or rose essential oils.

These findings, combined with ours study, underscore the potential of incorporating not only essential oils but also plant extracts with known antibacterial activity for the development of various bioactive textile materials. Plant extracts, similar to essential oils, are also known for their rich composition in bioactive compounds, though their specific constituents can vary widely depending on the plant source (Szadkowski et al. 2024). The literature highlights the potent antimicrobial effect of several plant extracts, including eucalyptus and hop (Kramer et al. 2015; Kolenc et al. 2022; Kunová et al. 2019; Zhou et al. 2019; Aleksic Sabo and Knezevic 2019). Numerous in vitro studies have demonstrated the antibacterial properties of different eucalyptus (Takahashi et al. 2004; Salari et al. 2006; Nasr et al. 2019) and hop (Kramer et al. 2015; Knez Hrncic et al. 2019; Kolenc et al. 2022; Weber et al. 2019), particularly against Gram-positive bacteria, including *S. aureus*, *Bacillus cereus*, *Enterococcus faecalis*, *Alicyclobacillus metagrophytes*. However, these extracts show limited efficacy against Gram-negative bacteria, such as *E. coli* and *Pseudomonas spp*.

The strong antibacterial activity of plant extracts against Gram-positive bacteria can be attributed to their simpler and more accessible cell wall structure. Despite having a thicker peptidoglycan layer, this structure allows bioactive compounds from plant extracts to penetrate and disrupt essential bacterial processes (Koohsari et al. 2015). In contrast, the complex outer membrane and additional protective mechanisms of Gram-negative bacteria offer robust defense, making them less susceptible to plant extracts. In addition, Gram-negative bacteria often employ strategies such as biofilm formation, creating a protective matrix that further shields them from antimicrobial agents. These biofilms make it even more challenging for the antimicrobial compounds in eucalyptus and hop extracts to penetrate and kill the bacteria, thereby reducing the effectiveness of these extracts against Gram-negative species.

Another important factor influencing this differential activity is the extraction process. Aqueous, ethanolic or hydro-ethanolic extraction may solubilize different plant components, which target Gram-positive and Gram-negative bacteria differently, as shown in previous works (Masoumian and Zandi 2017). In general, Gram-positive bacteria are more susceptible to phenolic compounds than Gram-negative bacteria. The outer membrane of Gram-negative bacteria provides a hydrophobic surface that excludes certain hydrophilic molecules, making them inherently resistant to many antimicrobial agents (Cueva et al. 2010). Gram-positive bacteria, lacking an outer membrane and enclosed in a plasma membrane covered by a thick peptidoglycan wall, are more vulnerable to these compounds (Alakomi et al. 2007). Although phenolic compounds are also effective against Gram-negative bacteria, their antimicrobial effect is more strain dependent (Patra 2012).

In summary, textiles functionalized with eucalyptus and hop extracts demonstrate a strong antibacterial effect against Gram-positive bacteria, making them suitable for treating skin infections caused by these bacteria. However, future research should focus on the stability and longevity of the antimicrobial effect of these textiles. In addition, testing a broader spectrum of bacteria, including more Gram-negative species and fungi, would provide a more comprehensive understanding of the antimicrobial potential of these plant extracts. This would help in determining the full range of effectiveness and possible applications for these bioactive textiles in different markets.

### Cytotoxicity evaluation of the knitted fabrics functionalized with eucalyptus and hop extracts

In addition to antibacterial activity, cytotoxicity is another crucial parameter that influences the application of any new textile material for industrial and medical purposes. Indeed, before these potential antimicrobial textile materials can be marketed, it is essential to thoroughly evaluate their biocompatibility and ensure they are safe for direct skin contact (Vojnits et al. 2024).

To assess the biocompatibility of knitted fabrics functionalized with plant extracts in this study, we employed three different cell culture assays to evaluate cytotoxicity in human skin cells. Given the close interaction between human skin and textile materials, we exposed keratinocytes in 24-well culture plates to direct contact with the different textile specimens. After 24 h, we assessed cell viability using both the MTT assay, TO/PI, and Calcein-AM/PI staining, key approaches for an initial in vitro screening.

To simulate prolonged usage conditions, such as hospital bedding where such products are typically changed within 24 h or between patient transitions, we selected a 24-h exposure time. This interval is sufficiently long to provide meaningful data without being influenced by other potential skin reactions such as irritations, infections, or unpleasant odors that could arise with longer exposure times (Stan et al. 2019). In addition, a 24-h exposure time also simulates one long sleep period in pyjamas, as the average sleep duration ranges from 7 to 9 h.

**Fig. 3:**
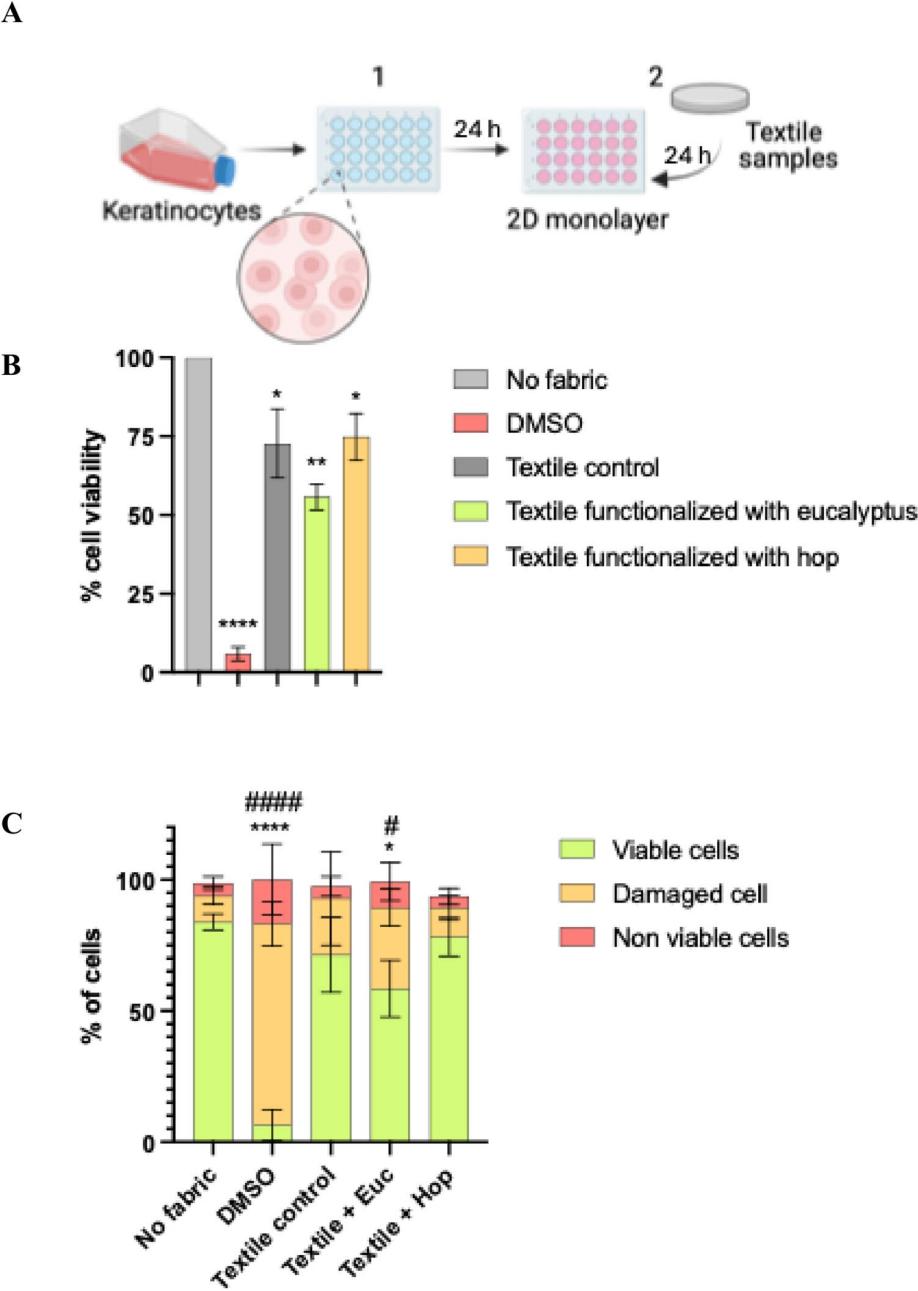
**A** Schematic representation of the direct contact method used to evaluate cell viability. **1** Keratinocytes were seeded in 24-well plates and incubated for 24 h. **2** Textile samples (1.5 cm^2^) were placed in each well. Cells cultured without fabric served as the negative control, while cells treated with 10% DMSO were used as the positive control. After 24 h of direct contact, cell viability was assessed using the MTT assay and TO/PI (thiazole orange/propidium iodide) staining. **B** Keratinocytes viability percentage from MTT assay are shown. Data represent the mean of three independent experiments. * indicates statistically significant differences compared to the negative control (no fabric). **C** Quantification of viable, damaged, and non-viable keratinocytes based on TO/PI staining. Data represent the mean of three independent experiments. * indicates statistically significant differences compared to viable cells of negative control (no fabric); # indicates statistically significant differences compared to damaged cells of negative control (no fabric)

After 24 h of incubation with the knitted fabrics, cell viability was assessed by measuring metabolic activity of human keratinocytes using the MTT assay (Fig. 3b). Compared to the negative control (cells cultured without fabric), cells exposed to 10% DMSO (positive control) exhibited significantly reduced cell viability activity (~ 10%; *p* < 0.0001), confirming its cytotoxic effect on keratinocytes. Among tested fabrics, both textile control and the textile functionalized with hop extracts demonstrated similar rates of cell viability (~ 75%; *p* < 0.05), suggesting biocompatibility. In contrast, the fabric functionalized with eucalyptus extract showed a slightly cell viability (~ 60%; *p* < 0.01), indicating moderate cytotoxicity (Fig. 3b).

Although there was a statistically significant difference between the textile control and both functionalized knitted fabrics (*p* < 0.05 and *p* < 0.01) compared to the negative control (cells cultured without fabric), this reduction is not considered toxic for biomedical devices, including functionalized textiles, according to ISO 10993-5 standards. Besides, it is known that incubating cells in direct contact with textiles without inserts present key disadvantages that may affect cell viability. For instance, cells can stick to the textiles during incubation, the textiles sitting on top of the cells can apply pressure affecting their normal spreading, and handling the textile in the well and plate can cause the detachment of several cells (Stan et al. 2019).

**Fig. 4:**
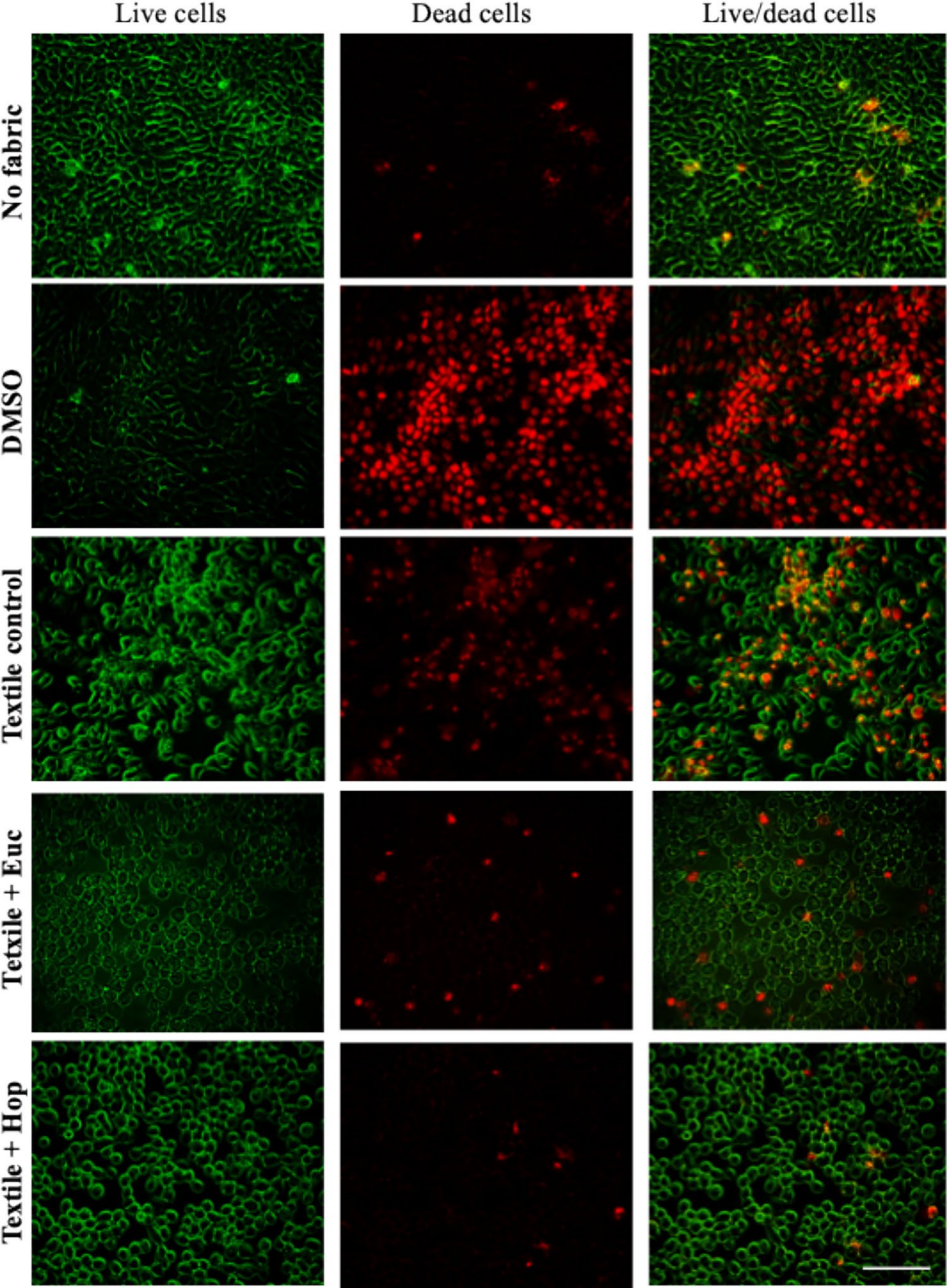
Representative images of live and dead keratinocytes after 24 h of direct contact with knitted fabrics functionalized with plant extracts. Keratinocytes were cultured in direct contact for 24 h with non-functionalized fabrics (textile control) or fabrics functionalized with eucalyptus and hop extracts. Cells were stained with Calcein-AM (live cells, green) and propidium iodide (PI; dead cells, red). Cells cultured without fabric served as the negative control, while those treated with 10% DMSO were used as the positive control. Scale bar: 200 μm

To complement these findings, we performed TO/PI, and Calcein-AM staining to assess keratinocyte viability in the presence of the functionalized knitted fabrics (Figs. 3c and 4). The proportion of viable, damaged, and non-viable keratinocytes was quantified after 24 h of incubation using TO/PI staining (Fig. 3c). Compared to the negative control (cells cultured without fabric), the textile control exhibited a high percentage of viable cells (~ 75%), with low levels of damaged cells (~ 20%) and non-viable cells (~ 5%), indicating good biocompatibility. Similarly, the fabric functionalized with hop extract maintained a high proportion of viable cells (~ 70%) and comparable proportions of damaged (~ 25%) and non-viable (~ 5%) cells, supporting its favorable biocompatibility (Fig. 3c).

In contrast, exposure to the eucalyptus-functionalized fabric showed a slightly lower percentage of viable cells (~ 65%; *p* < 0.05) and a moderate increase in damaged cells (~ 30%; *p* < 0.05), while non-viable remained low (~ 5%), indicating a moderate cytotoxicity profile with mild cellular stress. As expected, the positive control (10% DMSO) showed a drastic reduction in viable cells (~ 10%; *p* < 0.0001), with a marked increase in damaged (~ 75%; *p* < 0.0001) and non-viable (~ 15%) cells, confirming its strong cytotoxic effect.

To further complement these results and to visualize the cell morphology of the keratinocytes, a live dead assay (Calcein-AM/PI) through fluorescence microscopy was performed (Fig. 4). The images revealed clear differences in keratinocyte viability across the tested conditions. In the negative control (cells cultured without any fabric), keratinocytes formed a dense monolayer with predominantly green fluorescence (live cells) and minimal red fluorescence (dead cells), indicating high viability and healthy morphology. In contrast, the positive control (10% DMSO) exhibited extensive red stained cells with a marked loss of green signal, confirming strong cytotoxicity.

Cells exposed to the textile control exhibited mostly green fluorescence with a few scattered red cells, indicating good biocompatibility and minimal impact on cell morphology. Similarly, keratinocytes in contact with fabric functionalized with hop extract predominantly displayed green-stained cells, with a slight presence of red staining, indicating preserved viability accompanied by modest morphological changes (Fig. 4). In the case of the eucalyptus-functionalized fabric, cells exhibited a noticeable reduction in green-stained cells and increased red-stained cells, accompanied by pronounced cell morphological alterations, suggestive of cellular stress or damage. This is indicative of moderate cytotoxicity, probably associated with the (concentration) bioactive compounds present in the eucalyptus extract.

Together, these results confirm that knitted fabrics functionalized with some plant extracts, namely hop extracts are biocompatible and suitable for direct contact with human skin. Considering the market’s demand for bioactive textiles with antimicrobial activity, it is crucial to provide non-toxic products for daily human use. Although most antimicrobial textiles developed to date have demonstrated biocompatibility, many are impregnated with nanoparticles such as copper oxide, silver, or titanium dioxide (Vakayil et al. 2021; Stan et al. 2016; Singh et al. 2017), which were designed for industrial and commercial applications due to their potent bactericidal activity.

In contrast, to the best of our knowledge, few studies have prioritized the functionalization of textiles with natural compounds, namely plant extracts (Stan et al. 2019; Vakayil et al. 2021; Szadkowski et al. 2024). Unfortunately, current research studies involving textile materials with incorporation of plant extracts and/or essential oils have mostly focused on the antimicrobial activity of such products, being less concerned about the biocompatibility of the textile materials in contact with human skin tissues (Szadkowski et al. 2024; Kramar et al. 2021; Zaharia et al. 2020). However, a noteworthy study in this area highlighted different biocompatibility outcomes between direct and indirect contact cell culture of dermal fibroblasts when using textiles functionalized with microcapsules containing essential oils (Stan et al. 2019). Like our study, this research underscores the importance of exploring natural compounds for textile functionalization to ensure both efficacy and safety for daily human use while providing eco-friendly products.

## Conclusion

Our study highlights the promising potential of plant extracts, specifically from eucalyptus and hop, in developing bioactive textiles with antimicrobial activity. The knitted fabrics functionalized with eucalyptus and hop extracts exhibited strong antimicrobial activity against Gram-positive bacteria, including *Staphylococcus aureus* and *Staphylococcus epidermidis*. However, their effect on Gram-negative bacteria, namely *Escherichia coli*, was less pronounced.

In addition, biocompatibility assessments using two-dimensional (2D) human skin cells indicate that these textiles, especially those functionalized with hop extracts, show good biocompatibility for direct skin application. However, further investigations employing three-dimensional (3D) skin models are essential to provide a more comprehensive understanding of their effects. In addition, to ensure safety and regulatory compliance, it is also critical to conduct additional studies evaluating skin sensitization and genotoxicity, as these are key endpoints for assessing potential adverse effects on human health.

These findings support the utilization of plant extracts as natural antibacterial agents in textile applications, offering a sustainable and eco-friendly alternative to synthetic antimicrobials.

In summary, incorporating plant extracts in textile functionalization provides a valuable approach to creating antimicrobial materials that are both effective and safe for human use. Further research and development in this area are imperative to produce innovative products that meet the growing market demand, optimize their applications, and drive their ideal usage.

## Data Availability

All the data are available in the main text. In addition, any data in this study can be obtained from the corresponding authors upon reasonable request.
